# AGING IN PLACE WITH A WARMING CLIMATE: HOUSING POLICY AND DESIGN FOR AGING WITH EXTREME HEAT

**DOI:** 10.1093/geroni/igae098.3786

**Published:** 2024-12-31

**Authors:** Nichole Kain

**Affiliations:** Antioch University New England, Saint Paul, Minnesota, United States

## Abstract

Aging populations—particularly in areas prone to extreme heat—are facing unique residential challenges. In the past 10 years, extreme heat has killed more people in the United States (U.S.) than all other weather hazards combined, and it is expected to get hotter, with extreme heat events predicted to happen more often in the future. People over the age of 65 are disproportionately represented as the majority of heat victims, and are likely to perish in their own homes during these events. This study reveals that age-adaptive and climate-adaptive housing designs contribute to our quality of life and ability to survive as we age in a warming world. In addition to advancing our understanding of aging in places with extreme heat, this research offers two pieces of applied materials: A residential guide/handout for the general public that combines mitigation for both extreme heat and age-friendly design, as well as a policy research brief promoting the future development of energy efficient and age-friendly housing.

